# Antibiotic Use for Febrile Children in a Tertiary Care Hospital’s Outpatient Department: A Cross-Sectional Study

**DOI:** 10.7759/cureus.40356

**Published:** 2023-06-13

**Authors:** Yumna Asmaa, Spenta Kakalia, Muhammad Irtza, Rahat Malik, Muqaddas Jamshaid, Huma Farrukh

**Affiliations:** 1 Department of Paediatrics, Combined Military Hospital Lahore, Lahore, PAK; 2 Department of Paediatrics, Combined Military Hospital Lahore Medical College and Institute of Dentistry, Lahore, PAK; 3 Medical School, Services Institute of Medical Sciences, Lahore, Lahore, PAK; 4 Department of Paediatrics, MedEast Hospital, Lahore, PAK

**Keywords:** viral, bacteria, infection, respiratory tract, outpatient, pakistan, children, fever, antibiotics

## Abstract

Background

Irrational prescription of antibiotics is contributing to the antimicrobial resistance crisis in low and middle-income countries. Antibiotic stewardship programs need to be implemented to rationalize the use of antibiotics, but data on antibiotic prescriptions in pediatric outpatient departments is minimal. This study aimed to determine the frequency of antibiotic prescriptions in febrile children attending the Paediatric Outpatient Department (OPD) at Combined Military Hospital, Lahore, and observe the factors affecting the decision to prescribe antibiotics.

Methodology

A cross-sectional, descriptive study with non-probability sampling in the Department of Paediatrics at the Combined Military Hospital (CMH), Lahore, was conducted over two years. The confidence limit was 95%, and the anticipated population proportion was 32%. The primary outcome was the proportion of children aged two months to 10 years presenting to the OPD with fever who received antibiotics. Further analysis included the effect of patient-level risk factors on antibiotic prescription, especially in children with respiratory tract infections (RTIs).

Results

Of the 225 children analyzed, 137 (61%) received antibiotics. Of these antibiotic prescriptions, 123 (90%) were second-line antibiotics. Older age (odds ratio (OR) = 2.3, 1.18-4.46), high fever (OR = 2.48, 1.37-4.5), presenting in autumn and winter seasons (OR = 2.85, 1.53-5.3), ill appearance (OR = 2.71, 1.12-6.55), tachycardia (OR = 4.28, 1.22-15.01), and tachypnea (OR = 4.01, 1.14-14.12) were associated with increased likelihood of antibiotic prescription. Antibiotic prescriptions in children with RTIs were associated with lower RTI (OR = 12.96, 3.49-48.08), probable bacterial infection (OR = 12.37, 4.77-30.05), tachycardia (OR = 10.88, 1.28-92.24), tachypnea (OR = 14.73, 3.14-68.99), and increased work of breathing (OR = 7.8, 2.05-29.56).

Conclusions

The evidence of the widespread inappropriate use of antibiotics in OPDs, particularly for upper RTIs, highlights the need for an antibiotic stewardship program. Antibiotic overprescription promotes antibiotic resistance, prolonging illness and increasing healthcare costs.

## Introduction

The discovery of penicillin in 1928 started the golden age of antibiotics, resulting in cures for common infections and an increase in lifespan by 23 years [1]. However, since the mid-1950s, a gradual decline in antibiotic discovery and the development of antibiotic resistance in many common infectious pathogens has led to the current antimicrobial resistance crisis [2]. Antibiotics are among the drugs most prescribed to children in hospitals, the most common reason being fever [3]. Children suffering from viral infections or non-infectious diseases receive antibiotics frequently [4]. Antibiotic stewardship programs for pediatric patients are implemented across developed and developing countries to curb the burgeoning antibiotic resistance crisis [5,6].

There is evidence of irrational prescription of antibiotics in febrile children [3], especially with respiratory tract infections (RTIs) [7]. In low and middle-income countries (LMICs), high levels of antimicrobial resistance correlate with the high number of antibiotics prescribed in children with fever. Globally, the prevalence of antibiotic prescription is 32% [7], whereas, in a point prevalence multicenter study done in Punjab, Pakistan, the prevalence was 77% [8]. There is a need for more information about antibiotic prescriptions in pediatric outpatient departments (OPDs) as well as the factors contributing to increased antimicrobial medication. In Pakistan, minimal data are available and centered on specific areas, making it challenging to draw conclusions [9]. For successful implementation of interventions from antibiotic stewardship programs, it is crucial to have data about antibiotic prescriptions in the pediatric population and factors influencing the prescription of antibiotics in children with fever.

This cross-sectional study aimed to determine the frequency of antibiotic prescriptions for children presenting with fever in a pediatric OPD and whether the duration of the fever, the patient’s clinical condition, or the focus of infection affected the decision to prescribe antibiotics.

## Materials and methods

Study design and participants

A cross-sectional, descriptive study was conducted in the Department of Paediatrics at the Combined Military Hospital (CMH), Lahore, over two years from July 1, 2020, to June 30, 2022. The research and ethics board of CMH Lahore Medical College approved the study (approval number: 422/ERC/CMH/LMC). Children between the ages of two months and 10 years presenting to the OPD at CMH Lahore with fever as their primary complaint were included in the study. Exclusion criteria included children who had chronic or congenital neurodevelopmental, muscular, respiratory, or cardiac conditions; children who had visited the pediatric OPD multiple times in the past seven days with similar complaints; children who had used antibiotics in seven days before their visit; and children with an antibiotic allergy.

Procedure

The Raosoft® sample size calculator was used to calculate the sample size, keeping the confidence interval at 95% and the margin of error at 5% with an anticipated population of 32%. The estimated sample size was 329. Sampling days were scheduled randomly one day per week from 8 a.m. to 3 p.m. Data were collected from all children meeting the inclusion criteria who visited the department on the assigned sampling day to avoid selection bias. Data collection was done by researchers and appointed data collectors who were not directly involved in patient care by using the non-probability sampling technique. The data collectors filled out a structured printed questionnaire after obtaining verbal informed consent from the parents/caregivers at the end of their visit. SPSS Statistics for Windows, Version 26.0 (IBM Corp., Armonk, NY, USA) was used for data analysis. The authors followed the Strengthening the Reporting of Observational Studies in Epidemiology guidelines to report this study.

Outcome measures

The study’s primary outcome was the proportion of children who received antibiotics on visiting the OPD. The antibiotics were further analyzed by grouping them into first-line antibiotics (amoxicillin, narrow-spectrum penicillin, first-generation cephalosporins, and erythromycin) and second-line antibiotics (broad-spectrum penicillin, second and third-generation cephalosporins, trimethoprim-sulfamethoxazole (TMP-SMZ), macrolides (except erythromycin), aminoglycosides, fluoroquinolones, vancomycin, and metronidazole) [7].

Statistical analysis

**Figure 1:**
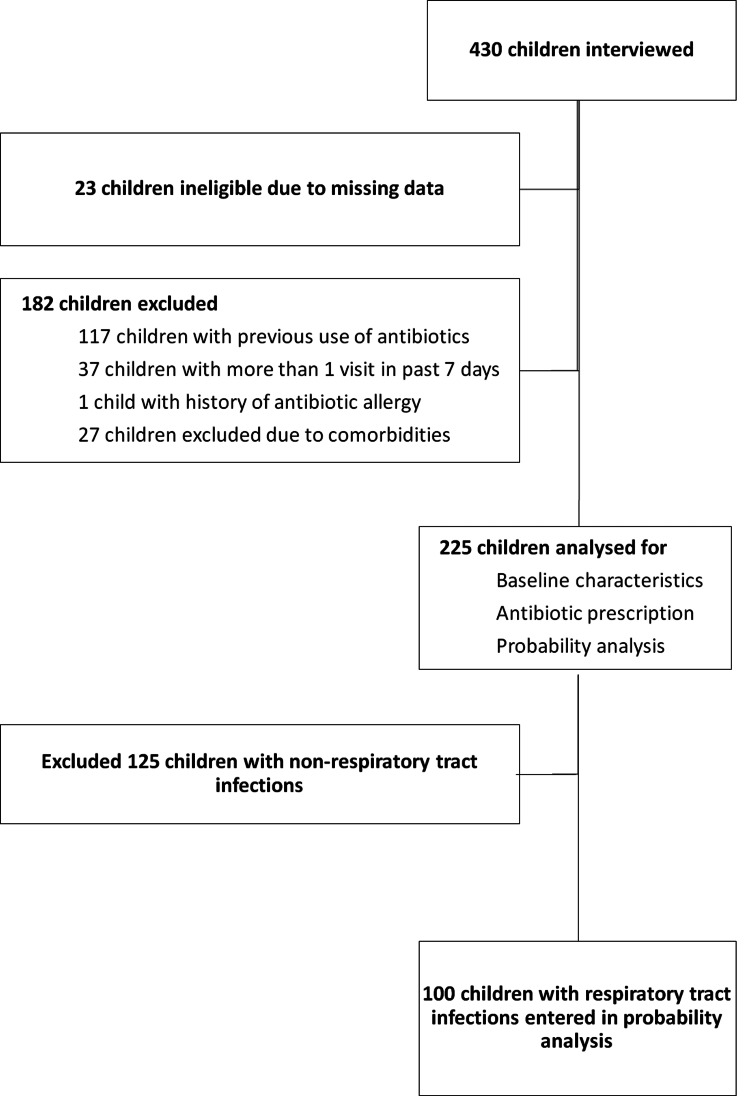
Study profile.

Of the 430 children, 23 children with missing data and 182 children meeting the exclusion criteria were excluded. The descriptive analysis of 225 children measured frequencies of patient characteristics and clinical factors, including the focus of infection and probable cause of illness (Figure 1). Aspects of patient management, including the number and type of antibiotics prescribed, were also analyzed. The additional analysis included the frequency of children who received antibiotics for each focus of infection.

For further analyses, variables were regrouped into two subcategories as follows: age of two months to five years and more than five years; duration of fever of fewer than three days and three days or more; a temperature of less than 100.5°F and 100.5°F or above; and the suspected cause of infection as viral and bacterial. The effect of patient-level risk factors for severe bacterial infection according to guidelines, including age, gender, fever duration, ill appearance, temperature, tachycardia, tachypnea, decreased consciousness, increased work of breathing, petechiae, signs of meningeal irritation, the focus of infection, and the season of the OPD visit on antibiotic prescription, was analyzed by calculating the odds ratio (OR). The influence of patient-level determinants on antibiotic prescriptions in children with RTIs was also determined by calculating the OR. For this analysis, children with another source of infection were excluded. All factors mentioned above were analyzed, and the results were combined.

## Results

**Figure 2:**
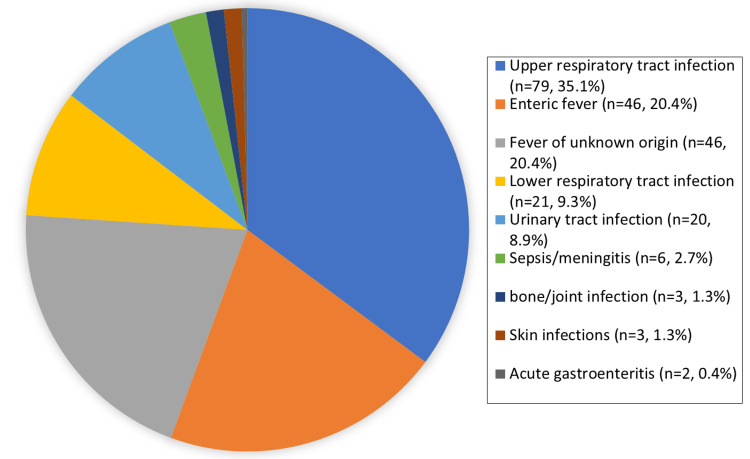
Frequency of the probable focus of infection. For complete data, see Table 4 in the appendix.

**Figure 3:**
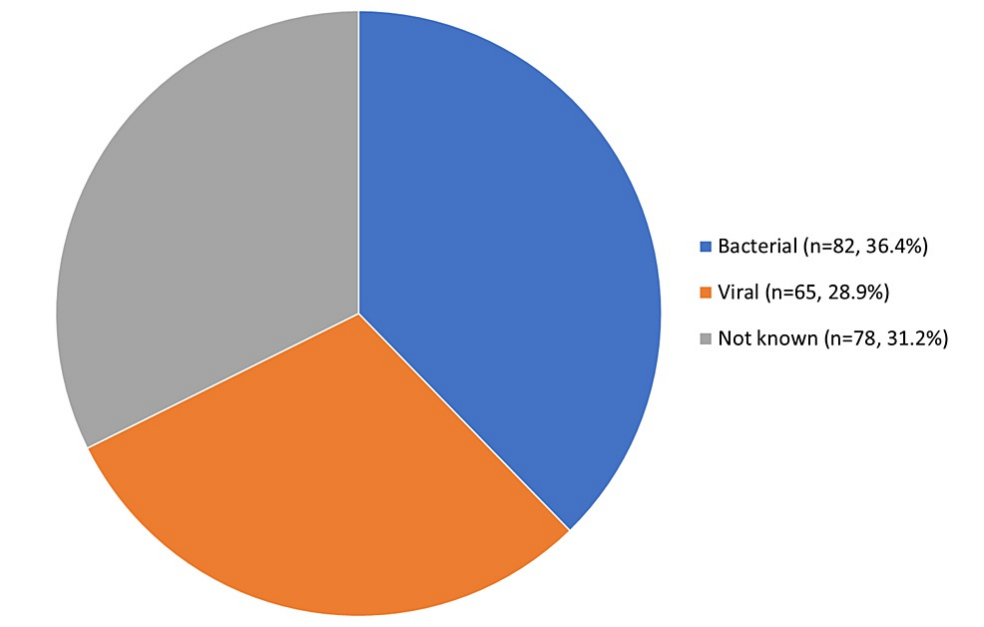
Frequency of probable cause of infection. For complete data, see Table 5 in the appendix.

A total of 225 children from pediatric OPD were included in the analysis of baseline characteristics, as summarized in Table 1. Most (42.7%) were between the ages of two months and two years, 13.8% were undernourished (weight for age less than the third centile), and 56.4% were male. Most of them (95%) were reviewed by the postgraduate trainee on arrival and supervised by the consultant pediatrician on the floor. The most common focus of infection was upper RTI (35.1%), followed by enteric fever (20.4%) and fever of unknown origin (20.4%) (Figure 2). The probable cause of infection was most often reported as bacterial (48.2%) (Figure 3). Overall, 61.3% of children were advised relevant cultures.

**Table 1:** Baseline characteristics of patients. ^a^: Unless stated otherwise;^ b^: defined as per the WHO guidelines (weight for age <-3SD); ^c^: these clinical parameters were defined as per advanced pediatric life support guidelines. n: proportion of patients; IQR: interquartile range; SD: standard deviation; GCS: Glasgow Coma Scale; ICU: intensive care unit

Parameters	Proportion of patients, n (%)^a^
General characteristics	
Age	
2 months to 2 years	96 (42.7%)
2 to 5 years	70 (31.1%)
5 to 10 years	59 (26.2%)
Underweight^b^	31 (13.8%)
Gender	
Male	127 (56.4%)
Female	98 (43.6%)
Season	
Spring	23 (10.2%)
Summer	53 (23.6%)
Autumn	30 (13.3%)
Winter	119 (52.9%)
Prescription by	
Consultant	11 (5%)
Postgraduate trainee	214 (95%)
Clinical condition	
Fever (days) (median + IQR)	3 (1–4)
Temperature (°F) (mean ± SD)	99.9 (1.66)
Ill appearance	33 (14.7%)
Tachycardia^c^	21 (9.3%)
Tachypnea^c^	20 (9%)
Increased work of breathing	17 (7.6%)
Petechiae	2 (1%)
Signs of meningeal irritation	4 (2%)
Delayed capillary refill time (>2 seconds)	1 (0.4%)
Altered mental status (GCS <12)	4 (2%)
Management	
Cultures advised	138 (61.3%)
Antibiotics prescribed	137 (61%)
Type of antibiotic prescribed	
First-line antibiotics	14 (10%)
Second-line antibiotics	123 (90%)
The number of antibiotics prescribed	
Single	125 (55.6%)
Multiple	12 (5.3%)
Conclusion of visit	
Outdoor prescription	121 (53.8%)
Observation for <24 hours	5 (2.2%)
Admitted to the ward	81 (36%)
Admitted to ICU	18 (8%)

During their OPD visit, 137 (61%) children received an antibiotic prescription, of which 123 (90%) were second-line antibiotics. Second and third-generation cephalosporins were among the most prescribed antibiotics (105 (70%)) (Table 2). Only 12 (5.3%) children received a combination of antibiotics, the most common being a carbapenem and vancomycin for sepsis/meningitis in three (25%) children (Table 2).

**Table 2:** Frequency of antibiotics prescribed to children.

Antibiotic class	Number of prescriptions, n (%)
Single antibiotic prescriptions	
Amoxicillin	12 (8%)
Broad-spectrum penicillin	3 (2%)
First-generation cephalosporins	9 (6%)
Second and third-generation cephalosporins	105 (70%)
Fluoroquinolones	6 (4%)
Clarithromycin	3 (2%)
Azithromycin	2 (1.5%)
Aminoglycosides	2 (1.5%)
Carbapenems	3 (2%)
Vancomycin	4 (3%)
Combinations of antibiotics prescribed	
Second and third-generation cephalosporins + azithromycin	2 (17%)
Second and third-generation cephalosporins + clarithromycin	2 (17%)
Second and third-generation cephalosporins + broad-spectrum penicillin	2 (17%)
Second and third-generation cephalosporins + aminoglycosides	2 (17%)
Second and third-generation cephalosporins + vancomycin	1 (8%)
Carbapenems + vancomycin	3 (25%)

**Figure 4:**
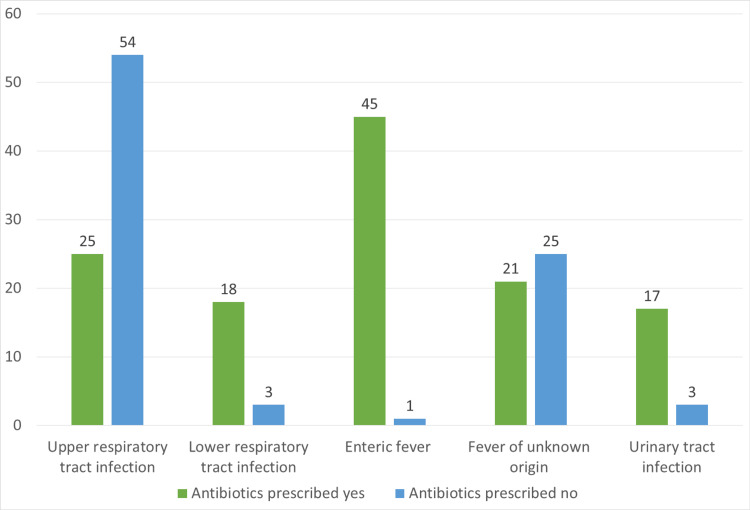
Frequency of antibiotic prescription for common foci of infection. For full data, see Table 6 in the appendix.

The five most common foci of infection were analyzed in the proportion of children receiving antibiotics according to the focus of infection, as summarized in Figure 4. The children with presumed enteric fever received antibiotics most frequently (45 (97.8%) of 46 children).

Children of older age, with higher temperatures (100.5°F or above) and presenting in autumn and winter seasons, were more likely to receive antibiotics. Other significant predictors included ill appearance, tachycardia, and tachypnea (Table 3).

**Table 3:** Analysis of factors affecting antibiotic prescription in all children with fever (a) and in children with respiratory tract infection (b). ^a^: standardized value; ^b^: significant predictor in all children with fever; ^c^: significant predictor in all children with fever as well as in children with RTIs; ^d^: Significant predictor in children with RTIs. RTI: respiratory tract infection; URTI: upper respiratory tract infection; LRTI: lower respiratory tract infection

Patient characteristics	a: All children who were prescribed antibiotics (odds ratio (95% CI))	b: Children with RTI who were prescribed antibiotics (odds ratio (95% CI))
Age^a, b^	2.3 (1.18-4.46)	0.572 (0.176-1.722)
Weight	0.83 (0.37-1.84)	0.731 (0.198-2.704)
Gender	0.904 (0.526-1.553)	0.815 (0.362-1.835)
Season^a, b^ (autumn-winter/spring-summer)	2.85 (1.53-5.3)	0.462 (0.160-1.335)
Ill appearance^b^	2.71 (1.12-6.55)	2.25 (0.73-6.89)
Tachycardia^c^	4.28 (1.22-15.01)	10.88 (1.28-92.24)
Tachypnea^c^	4.01 (1.14-14.12)	14.73 (3.14-68.99)
Respiratory distress^c^	3.22 (0.89-11.56)	7.8 (2.05-29.56)
Duration of fever (days)^a, b^	1.58 (0.92-2.71)	0.8 (0.36-1.77)
Temperature (°F)^a, b^	2.48 (1.37-4.5)	2.22 (0.915-5.39)
The suspected cause of infection^a, c^	0.055 (0.026-0.116)	12.37 (4.77-30.05)
The focus of infection (URTI vs. LRTI)^d^		12.96 (3.49-48.08)

Of children diagnosed with RTIs (upper respiratory tract infections (URTI) and lower respiratory tract infections (LRTI)), 43% received antibiotics (43 out of 100). LRTI and probable bacterial infection increased the likelihood of antibiotic prescription in these children. Other significant factors for antibiotic prescription included tachycardia, tachypnea, and increased work of breathing (Table 3).

## Discussion

This study revealed frequent use of antibiotics in children in OPDs (61%), which was relatively higher than the previously reported international studies [7] but lower than the local studies [8]. The most significant predictors of antibiotic prescription were older age and high-grade fever.

In this study, the most common cause of fever was URTI (35.1%), followed by enteric fever (20.4%) and fever of unknown origin (20.4%). A systemic review examined causes of fever in LMICs and concluded that 17.9% of febrile patients had test results positive for at least one pathogen. Of these, 10.3% had blood culture-confirmed bacterial or fungal infections, 28.5% had malaria, and 17.4% had viral infections [10]. The studies included in this review were from many countries, confirming the heterogeneity in pathogens causing fever. This study did not include febrile patients seen in emergency rooms (ERs) or OPDs, which might be the reason why respiratory pathogens were not reported, demonstrating that pathogens for fever are geographically diverse, underscoring the importance of local data for causes of fever.

The present study highlighted increased prescription of second-line antibiotics (90%), with a preponderance of second and third-generation cephalosporins. In Melbourne, less than 3% of the children were prescribed first-line antibiotics such as amoxicillin [11]. In a point-prevalence study, TMP-SMZ was the most prescribed antibiotic in Japan [12]. In another study in India, aminoglycosides and third-generation cephalosporins were most used for respiratory infections and acute febrile illnesses [13]. The evidence confirms that antimicrobial stewardship needs to be followed appropriately in the OPD setting.

In this study, the most common reason for antibiotic prescription was presumed enteric fever (in 45 out of 46 children (97.8%)). Due to the emergence of extremely drug-resistant (XDR) typhoid, with sensitivity to only azithromycin and meropenem, there is a shift in antibiotic prescription trends [14]. Audits of antibiotics used empirically for enteric fever demonstrate the extensive use of third-generation cephalosporins, an antibiotic recommended in Pakistan as empiric treatment [15,16]. Examining the current antibiogram for isolates at the institute where this study was performed (from January 1, 2022, to June 30, 2022), most bacterial isolates from the pediatric inpatient unit, including more than 50% *Salmonella typhi* (13 out of 21), were sensitive to ceftriaxone. From this antibiogram, it is justified to start third-generation cephalosporins for suspected enteric fever after sending blood cultures. However, when the overall first-choice antibiotic selection in the studied population was analyzed, the study found that most children with fever, especially RTIs, were prescribed second or third-generation cephalosporins. Third-generation cephalosporins, especially ceftriaxone, are on the watch list in the WHO AWaRe (Access, Watch, Reserve) program, a concern for increasing antimicrobial resistance to these drugs [17].

The main strength of this study is that it provides insights into the factors affecting the decision to prescribe antibiotics. Factors that positively correlated with receiving a prescription for an antibiotic were age (older age), season (fall and spring), ill appearance, tachycardia, tachypnea, respiratory distress, high temperature, and suspected bacterial infection. This is consistent with previously reported predictors of severe bacterial infection [7,18]. However, these drivers can only explain a part of the reason for the increased antimicrobial prescription.

In children presenting with URTI, 31.6% (25 out of 54) received antibiotics. Khan et al. observed the response of only supportive care in children with URTI. Of these, 76% recovered after one week, 16% eventually received oral antibiotics, and 4% were hospitalized [19]. Van de Matt et al. explored antibiotic prescriptions for fever in European countries, and the results demonstrated 16% to 67% prescriptions for URTIs and 24% to 87% for LRTIs [7]. In India, Rajeswani et al. found that URTI was the main reason for prescribing antibiotics in a pediatric OPD [20]. A large study in England discovered a common practice of prescribing antibiotics for cough and bronchitis (41.6%), sore throat (25.7%), acute otitis media (8.9%), and acute sinusitis (8.2%) [21]. This evidence confirms a trend for overuse of antibiotics to treat URTI, which usually responds to symptomatic treatment only [19].

Up to 85% (18 out of 21) of children with LRTIs received antibiotics. The increased prescription of antibiotics in LTRIs can be partially explained by the lack of a gold standard for diagnosing LRTIs. Viral panels can isolate the organism; however, they are expensive and only done in a few specialized laboratories [22]. A retrospective study in Punjab, Pakistan, examined the management of children hospitalized with LRTIs. Of these, 70.9% of children were diagnosed with community-acquired pneumonia, followed by acute bronchitis (14%). All these children received antibiotics, which were found to be inappropriate in 90% of cases [23].

In further analysis of antibiotic prescription in RTIs ((43 out of 100) 43%), tachycardia, tachypnea, signs of respiratory distress, LRTI, and probable bacterial infection increased the likelihood of an antibiotic prescription. Examining similar factors contributing to antibiotic prescription in febrile patients with RTIs, van de Matt et al. found correlations with age (older age), fever duration, C-reactive protein, chest radiography results, and focal abnormalities [7]. Karthikeyan et al., in their study in India, found that antibiotics were more likely to be prescribed to patients with higher temperature, younger age, males, living in a joint family, highly educated, and internet access [24]. A survey done in Punjab, the largest province in Pakistan, revealed that 34.5% of healthcare workers believed antibiotics to be the cure for viral infections, and 47.5% thought antibiotics were effective against the common cold and flu [25]. The inappropriate use of antibiotics promotes antimicrobial resistance. Therefore, antimicrobial stewardship intervention is essential to better control antibiotic use and combat antimicrobial resistance [26]. The evidence reflects a need to educate medical students, doctors, and other healthcare workers about preventing antimicrobial resistance, including public awareness; it also highlights the importance of antimicrobial stewardship programs in LMICs [27].

The study had some limitations: There needed to be a follow-up, resulting in a lack of knowledge about the outcome. RTIs include multiple diagnoses, such as acute otitis media, pneumonia, or bronchitis, each having different treatment regimens. This information was not included in this study; however, because the data were collected in all seasons for two years, the authors believe all diagnoses were equally represented. Although there was an antibiogram for inpatients, one for the OPD was unavailable. The authors have extensively corrected many known influential factors, believing the analysis yielded valid results. Finally, this was a single-center study with a small sample size. Given the heterogeneity of a city’s population, it is impossible to generalize results from a single center. The authors suggest a large multicenter study to observe the variability in antimicrobial prescription and the hospital factors for antimicrobial resistance.

## Conclusions

This study emphasizes the inappropriate use of antibiotics in pediatric OPDs for treating predominantly viral URTIs. The misuse of antibiotics poses a significant public health threat by promoting antibiotic resistance, prolonging illness, and increasing healthcare costs. Therefore, a concerted effort is necessary to implement effective antibiotic stewardship programs involving collaboration among healthcare professionals, including physicians, pharmacists, microbiologists, hospital administration, and public education. National and local guidelines are required for rationalizing antibiotic use based on local antibiograms. These actions can reduce unnecessary antibiotics, preserve their effectiveness, and protect public health.

## Appendices

**Table 4:** Frequency of the probable focus of infection.

Probable focus of infection	Frequency	Percent	Valid percent	Cumulative percent
Upper respiratory tract infection (n = 79, 35.1%)	79	35.1	25.8	25.8
Enteric fever (n = 46, 20.4%)	46	20.4	28.9	64.2
Fever of unknown origin (n = 46, 20.4%)	46	20.4	20.8	85
Lower respiratory tract infection (n = 21, 9.3%)	21	9.3	9.5	35.3
Urinary tract infection (n = 20, 8.9%)	20	8.9	8.7	93.7
Sepsis/meningitis (n = 6, 2.7%)	6	2.7	2.6	97.1
Bone/joint infection (n = 3, 1.3%)	3	1.3	2.4	99.5
Skin infections (n = 3, 1.3%)	3	1.3	0.8	94.5
Acute gastroenteritis (n = 2, 0.4%)	1	0.4	0.5	100

**Table 5:** Frequency of the probable cause of infection.

Suspected cause of infection	Percentage
Bacterial (n = 82, 36.4%)	36.40%
Viral (n = 65, 28.9%)	28.90%
Not known (n = 78, 31.2%)	31.20%

**Table 6:** Frequency of antibiotic prescription per foci of infection. ^a^: includes children suffering from upper respiratory tract infections and lower respiratory tract infections. n: proportion of children with each focus of infection

Focus of infection	Antibiotics prescribed		Percentage
	Yes	No	
Upper respiratory tract infection (n = 79, 35.1%)	25	54	31.60%
Enteric fever (n = 46, 20.4%)	45	1	97.80%
Fever of unknown origin (n = 46, 20.4%)	21	25	45.60%
Lower respiratory tract infection (n = 21, 9.3%)	18	3	85.70%
Urinary tract infection (n = 20, 8.9%)	17	3	85%
Sepsis/meningitis (n = 6, 2.7%)	6	0	100%
bone/joint infection (n = 3, 1.3%)	3	0	100%
Skin infections (n = 3, 1.3%)	2	1	66.70%
Acute gastroenteritis (n = 2, 0.4%)	0	2	
Respiratory tract infections^a^ (n = 100)	43	57	43%
